# Activation of WNT and BMP signaling in adult human articular cartilage following mechanical injury

**DOI:** 10.1186/ar2029

**Published:** 2006-08-07

**Authors:** Francesco Dell'Accio, Cosimo De Bari, Noha MF El Tawil, Francesca Barone, Thimios A Mitsiadis, John O'Dowd, Costantino Pitzalis

**Affiliations:** 1 Department of Rheumatology, King's College London, London, UK; 2 Department of Craniofacial Development, King's College London, London, UK; 3 Guy's and St Thomas's Hospitals, London, UK

## Abstract

Acute full thickness joint surface defects can undergo repair, which involves tissue patterning and endochondral bone formation. Molecular signals regulating this process may contribute to the repair outcome, chronic evolution and, eventually, the onset of osteoarthritis. We tested the hypothesis that mechanical injury modulates morphogenetic pathways in adult human articular cartilage explants. Adjacent articular cartilage explants were obtained from preserved areas of the femoral condyles of patients undergoing arthroplasty for osteoarthritis, or from a normal joint of a patient undergoing lower limb amputation. Paired explants were individually maintained in explant culture. From each pair, one explant was mechanically injured and the other left uninjured as a control. Cultures were terminated at different time points for histochemistry, immunohistochemistry and gene expression analysis by reverse transcription real time PCR. *Bone morphogenetic protein 2 *(*BMP-2*) mRNA was upregulated in the injured explants. We detected phosphorylation of SMAD-1 and SMAD-5, consistent with activation of the bone morphogenetic protein (BMP) pathway. *FRZB-1 *mRNA was downregulated in the injured explants, suggesting de-repression of WNT signaling. Accordingly, expression of the canonical WNT target genes *Axin-2 *and c-JUN was upregulated in the injured explants. Activation of the canonical WNT signaling pathway by LiCl treatment induced upregulation of *COL2A1 *and Aggrecan mRNA, suggesting an anabolic effect. Phosphorylation of SMAD-1/-5 and downregulation of FRZB were confirmed in vivo in a mouse model of joint surface injury. Taken together, these data show modulation of the BMP and WNT pathways following mechanical injury *in vitro *and *in vivo*, which may play a role in the reparative response of the joint surface. These pathways may, therefore, represent potential targets in protocols of biological joint surface defect repair.

## Introduction

Chronic symptomatic full thickness defects of the joint surface are commonly regarded to have a poor repair capacity. Therefore, surgical treatment is provided for symptomatic relief and in an attempt to avoid possible evolution towards osteoarthritis (OA) [1]. The natural history of acute full thickness joint surface defects (JSDs), however, is not yet well known. Scattered clinical and animal studies have suggested that acute full thickness JSDs exhibit potential for repair, which is dependent on age, the size of the lesion, and biomechanical factors.

In two independent, long term, prospective studies, acute traumatic chondral lesions in young athletes had a good to excellent clinical outcome in 78% of the cases in the absence of specific surgical treatments [2,3]. In addition, Koshino and colleagues [4] reported significant regeneration of chronic JSDs associated with genu varu at 2 years after correction of knee malalignment by valgus osteotomy. Age dependent spontaneous repair has been reported in patients with osteochondritis dissecans [5]. Likewise, age dependent spontaneous repair of relatively small experimental full thickness JSDs has been reported in rabbits [6,7] and dogs [8]. In rabbits, this repair process entails invasion of the fibrin clot, filling the defect by mesenchymal progenitors, chondrogenesis, and endochondral bone formation. Bone formation is polarized towards the joint surface, and preserves a layer of articular cartilage [6]. Although the repair tissue is not always durable and advancement of the bone front at the expense of stable articular cartilage sometimes occurs, this repair process, under specific conditions, can restore joint surface homeostasis.

The patterning and morphogenesis that joint surface repair entails implies a stepwise cellular and molecular program. Thus, failure of the signaling mechanisms governing this process may be a factor contributing to a poor repair outcome. Such signals may represent therapeutic targets to support spontaneous repair or complement existing biological joint resurfacing techniques.

The current surgical approaches for localized full thickness lesions of the joint surface are autologous chondrocyte implantation, microfracture, and mosaicplasty. However, clinical outcomes suffer from some degree of variability [9-11]. In addition, there is still no satisfactory biological regeneration protocol for non-localized lesions. An alternative or complementary approach for joint tissue repair would be the controlled delivery of molecular signals to mesenchymal progenitors reported within the joint environment [12-18] with support of the subsequent steps of repair, including proliferation, patterning, and differentiation *in vivo*.

In this study, we have tested the hypothesis that the adult human articular cartilage is a source of morphogenetic signals upon injury. To this end, we have used an *in vitro *model of mechanical injury to the adult human articular cartilage to screen signaling pathways potentially involved in the repair response. In particular, we have focused on the bone morphogenetic protein (BMP) and the canonical WNT pathways, which are known to play a crucial role in joint morphogenesis and homeostasis as well as in repair processes [19-21].

BMPs are secreted molecules belonging to the transforming growth factor β superfamily of morphogens. Upon binding their ligands, BMP receptors phosphorylate the carboxy-terminal domain of SMAD-1, SMAD-5 and SMAD-8. Phosphorylated SMADS translocate to the nucleus where they participate in the transcriptional regulation of target genes [20].

WNTs constitute a large family of morphogens. WNT ligands transduce their signal through different intracellular pathways. In the β catenin-dependent (canonical) pathway, in the absence of WNT ligands, glycogen synthase kinase 3 (GSK-3) constitutively phosphorylates β catenin, which then is degraded through the proteasome pathway. When WNT ligands bind to their receptors (called FRZD), GSK-3 is inhibited and β catenin is, therefore, stabilized and accumulates in the cytoplasm and translocates into the nucleus, where it binds to members of the T-cell factor/lymphoid enhancer factor (TCF/LEF) family of transcription factors, thereby activating transcription of target genes [22].

## Materials and methods

### *Ex vivo *cartilage injury model and tissue culture

**Figure 1:**
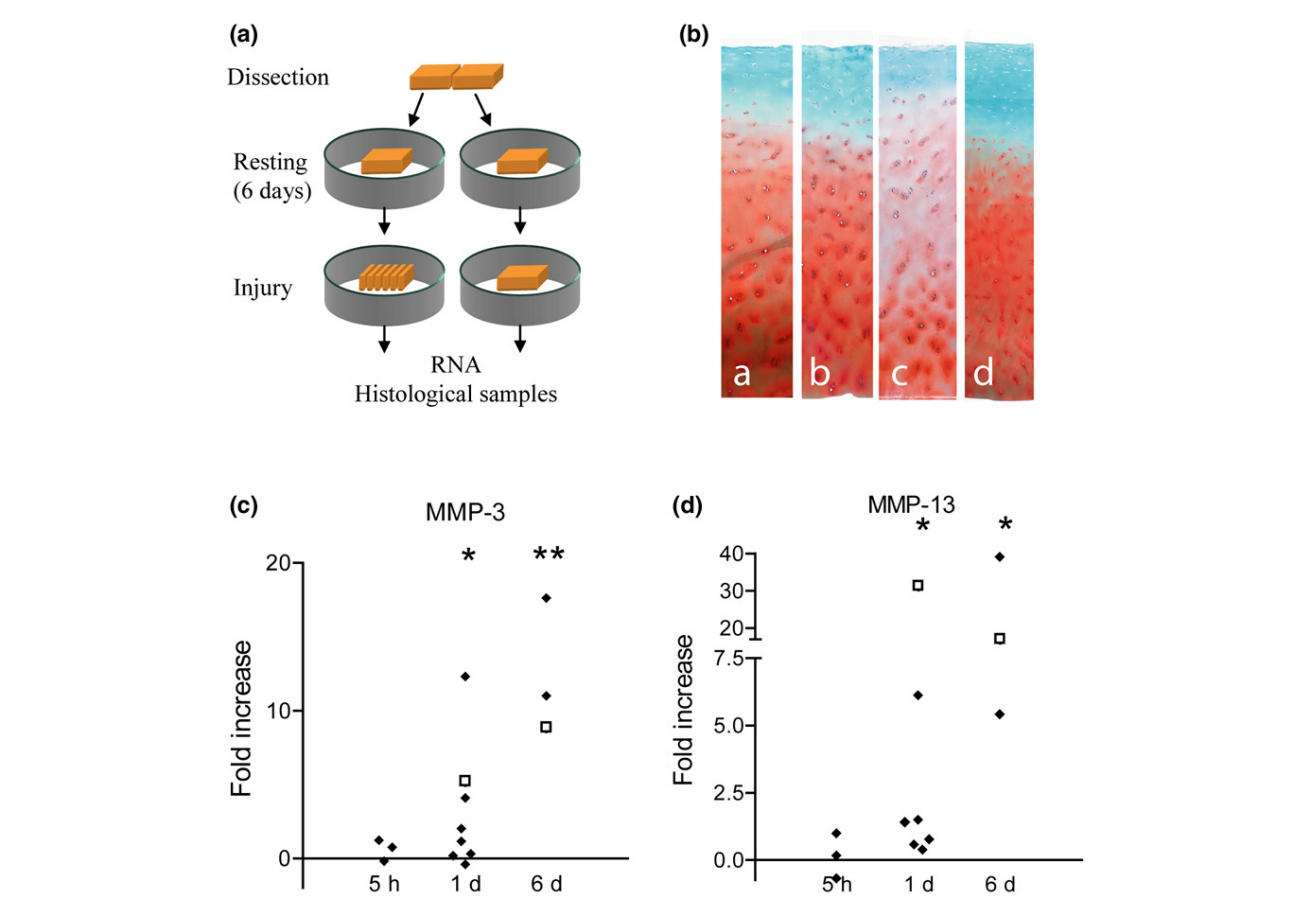
*Ex vivo *model of mechanical injury to adult human articular cartilage explants. **(a) **Adjacent explants from human adult articular cartilage were dissected and placed in culture in separate bacteriological Petri dishes. After 6 days, 1 explant was injured. At different time points the cultures were terminated for gene expression analysis, histochemistry and immunohistochemistry. **(b) **Safranin O staining of: a, freshly dissected normal articular cartilage; b, an adjacent explant after 7 days in culture; c, a further adjacent explant after 6 days in culture before injury plus 1 additional day after injury; and d, a typical freshly dissected explant from a preserved area from a patient who had undergone joint arthroplasty for osteoarthritis. **(c,d) **Time course of *metalloproteinase *(*MMP*)-*3 *and *MMP-13 *mRNA differential expression in injured versus uninjured explants. Values are normalized for the housekeeping gene *β actin *and expressed as fold change of gene expression in the injured explants from paired uninjured controls. Diamonds indicate samples from preserved areas from joints affected by osteoarthritis; open squares indicate sample pairs from healthy cartilage. *p < 0.05; **p < 0.01. D, day(s); h, hours.

Well-preserved (modified Mankin score 5 or less) cartilage samples were obtained from patients who underwent total knee replacement for unicompartmental OA (e.g., lateral condyle in genu varu). The average age was 67.5 ± 8.9 years old and the study included 3 males and 5 females. In one case (male, 49 years old), we obtained cartilage explants from a patient who had undergone limb amputation due to a road traffic accident and was free from OA. In this case, therefore, the cartilage was considered normal. Paired adjacent explants of approximately 6 × 6 mm were maintained in culture in 4 ml of Dulbecco's modeified Eagle's medium/HAMF12 1:1 (Invitrogen, Paisley, UK) in the presence or in the absence of 10% FBS (Invitrogen) and antibiotics/antimycotics (Invitrogen) in individual 33 mm bacteriological Petri dishes (BD Falcon™, BD Biosciences, Le Pont De Claix, France). We used bacteriological Petri dishes to avoid spreading of cells from the explants. After 6 days, the medium was replaced and one of each pair of adjacent samples was cut using a scalpel at 1 mm intervals. The other explant of each pair was left uninjured (Figure 1a). At different time points, the explants were used for RNA extraction and one aliquot was processed for histology and immunohistochemistry.

For experiments investigating activation of the WNT/β catenin canonical pathway by means of LiCl treatment, the explants were maintained for 6 days in complete culture medium containing 10 mM NaCl. At the end of this period, the explants were either switched to medium containing 10 mM LiCl or, for control explants, the medium was replaced with fresh medium containing 10 mM NaCl. The experiments were then terminated after one day. All procedures received approval from the local ethics committee.

### RNA extraction, reverse transcription PCR and quantitative real time RT-PCR

Cartilage samples were snap-frozen in liquid nitrogen, powdered with a mortar and pestle in liquid nitrogen, and subsequently homogenized in Trizol reagent (Life Technologies, Invitrogen, Paisley, UK) using a polytron homogenizer. Total RNA was extracted using Trizol reagent. Reverse transcription PCR (RT-PCR) was performed as described elsewhere [23]. Quantitative real time RT-PCR (Q-PCR) was performed using hot start DNA polymerase (Quiagen Ltd, Crawley, UK) in the presence of 0.1X SYBR Green (Molecular Probes, Invitrogen, Paisley, UK) utilizing the DNA Engine Opticon^® ^2 System (MJ Research, Alpha technologies Ltd, Northern Ireland). Reactions were performed in duplicate and repeated in the rare cases when the Ct of the duplicates differed for more than 1 cycle. A serial dilution of a cDNA from early passage human articular chondrocytes was used for a standard curve. Gene expression was calculated using a standard curve and normalized for the expression of the housekeeping gene β actin. To simplify the representation of time course analyses, the gene expression data normalized for β actin are shown as fold increase from uninjured paired control.

Primers and expected amplicon size are: *β-actin *(GeneBank:BC014861), forward 5'-CACGGCTGCTTCCAGCTC-3', reverse 5'-CACAGGACTCCATGCCCAG-3', 134 base pairs (bp); *MMP-3 *(GeneBank:NM_002422), forward 5'-CAACCGTGAGGAAAATCGATGCAG-3', reverse 5'-CGGCAAGATACAGATTCACGCTCAA-3', 440 bp; *MMP13 *(GeneBank:NM_002427), forward 5'-ACGGACCCATACAGTTTGAATACAGC-3', reverse 5'-CCATTTGTGGTGTGGGAAGTATCATC-3, 360 bp; *BMP-2 *(GeneBank:NM_001200), forward 5'-CGTCAAGCCAAACACAAACAGCG-3', reverse 5'- CACCCACAACCCTCCACAACCAT-3', 341 bp; *FRZB *(GeneBank:U24163), forward 5'GGGCTATGAAGATGAGGAACGT-3', reverse 5'-ACCGAGTCGATCCTTCCACTT-3', 79 bp; *β catenin *(GeneBank:X87838), forward 5'-CCAGCCGACACCAAGAAGCA-3', reverse 5'-GCGGGACAAAGGGCAAGATT-3', 151 bp; *WNT1 *(GeneBank:NM-005430), forward 5'-CTGCCTCTCTTCTTCCCCTT-3', reverse 5'-TCACAGCTGTTCAATGGCTC-3', 251 bp; *WNT5A *(GeneBank:L20861), forward 5'-CCACCTTCCTCTTCACACTG-3', reverse 5'-CGAACAAGTAATGCCCTCTC-3', 770 bp; *WNT5B *(GeneBank:AB060966), forward 5'- CCGCCTCTGCAACAAGACCT-3', reverse 5'- AACTTGCAGTGGCAGCGCTC-3', 111 bp; *WNT14 *(GeneBenk:NM_003395), forward 5'- TGAGAAGAACTGCGAGAGCA -3', reverse 5'- CTGTGTGCAATGCCTGTACC -3', 285 bp; *WNT16 *(GeneBank:NM_016087), forward 5'- AAAGAAATGTTTCCCTGCCC -3', reverse 5'- GACATTTTCCATGGGTTTGC -3', 106 bp; *FRZD-1 *(GeneBank:NM_003505), forward 5'- TTCAGCAGCACATTCTGAGG-3', reverse 5'- CCTGCACACATTTTCCCTTT-3', 154 bp; *FRZD-7 *(GeneBank:NM_003507), forward 5'- CTGGAGTTCTTTGAAATGTGCT-3', reverse 5'- AAGGTTAGCTCCCATGATTCTC-3', 133 bp; *LEF-1 *(GeneBank:NM_016269), forward 5'- CAGAGAAAGGAGCAGGAGCCAA -3', reverse 5'- TGATGTCAGTGTTCCTTTGGCG -3', 481 bp; *TCF-1 *(GeneBank:NM000545), forward 5'- CTCATCACCGACACCACCAACC-3', reverse 5'- TCCCACGAAGCAGCGACAGT -3', 608 bp; *COL2A1 *(GeneBank:NM_033150), forward 5'- CCCTGAGTGGAAGAGTGGAG -3', reverse 5'- GAGGCGTGAGGTCTTCTGTG -3', 511 bp; *Aggrecan *(GeneBank:NM-001135), forward 5'- GTTGTCATCAGCACCAGCATC -3', reverse 5'- ACCACACAGTCCTCTCCAGC -3', 509 bp; *c-JUN *(GeneBank:NM_002228), forward 5'-CCCCAAGATCCTGAAACAGA-3', reverse 5'- CCGTTGCTGGACTGGATTAT-3'.

### Histology, histochemistry and immunohistochemistry

Tissues were fixed overnight in 4% buffered paraformaldehyde at 4°C, dehydrated and embedded in paraffin. Sections (5 μm thick) were used for hematoxylin-eosin and safranin O staining according to standard protocols. The degree of OA was evaluated using a modified Mankin score [24] in which the sub-score related to the tide mark was not included. For immunohistochemistry, paraffin sections were deparaffinized and hydrated in xylene and an ethanol series, post-fixed with 4% paraformaldehyde, and washed twice in phosphate-buffered saline. For antigen retrieval in the detection of FRZB and β catenin, the sections were first equilibrated in 0.02% HCl for 7 minutes, digested in 3 mg/ml pepsin (Sigma-Aldrich Company Ltd., Gillingham, UK) in 0.02% HCl for 45 minutes at 37°C, washed in water and allowed to air dry for 20 minutes. Sections were washed twice in 0.2% Tween-20 in tris buffered saline (TBST), blocked in 0.5% bovine serum albumin in TBST for 1 hour at room temperature, blotted, and incubated overnight with the primary antibody (goat anti-mouse/human FRZB (R&D Systems, Abingdon, UK), or mouse anti-human β catenin (BD Transduction Laboratories, BD, Cowley, Oxford, UK) at a final concentration of 1 μg/ml in 0.5% bovine serum albumin in TBST. Sections were then washed twice in TBST, and incubated for 1 hour with the secondary antibody. For FRZB immunostaining, the secondary antibody was a biotin-conjugated rabbit anti-goat antibody (DAKO UK Ltd., Ely Cambridgeshire, UK) diluted 1:300. For β catenin immunostaining, we used either a cy™2 conjugated goat anti-mouse antibody (Jackson ImmunoResearch Laboratories, Inc. West Grove, PA, USA) diluted 1:200 for indirect immunofluorescence, or the StreptABComplex/AP kit (DAKO) for signal amplification and Vector^® ^Red substrate kit (Vector Laboratories UK, Peterborough, UK) as a chromogenic substrate of alkaline phosphatase, in the presence of 0.2 mM levamisole to inhibit endogenous alkaline phosphatase. For the detection of phosphorylated SMAD-1 and SMAD-5, we used the same protocol with the following modifications. For antigen retrieval, instead of pepsin digestion, we boiled the sections for 10 minutes in sodium citrate buffer, pH 6; we quenched endogenous peroxidase by incubating for 10 minutes with 9% H_2_O_2_; we used the PS-1 antiserum [25] (a kind gift of P ten Dijke and C-H Heldin, Ludwig Institute for Cancer Research, Uppsala, Sweden) as primary antibody; as secondary antibody we used biotin-conjugated sheep anti-rabbit antibody (Serotec UK, Oxford, UK) diluted 1:200; we used the StreptABComplex/AP kit (DAKO) as an amplification system, and Liquid DAB Substrate Chromogen System (DAKO) as peroxidase substrate. Sections were mounted in mowiol (Calbiochem, Merck Biosciences Ltd, Nottingham, UK) containing 49,6-diamidino-2-phenylindole (DAPI; ICN, Stretton Scientific Ltd., Stretton, UK) for nuclear counterstaining. In positive cells the DAB precipitate quenched the DAPI fluorescence. Image processing was performed using Adobe Photoshop version 6 (Adobe). Negative controls were sections in which isotype and species-matched non-specific immunoglobulins or normal rabbit serum (for phospho-SMAD-1/-5) were used instead of the primary antibody.

### Statistical analysis

Normally distributed data sets from paired samples were compared using the paired *t *test. When the values did not have a normal distribution, they were either transformed into their logarithms before analysis or, if this still did not result in a normal distribution, they were analyzed using the Wilcoxon matched pair test.

### Joint surface injury in mice

Seven week old C57BL/6 male mice were utilized for these experiments. The mice were anesthetized and subjected to medial para-patellar arthrotomy. The patellar groove was exposed by lateral patellar dislocation. A longitudinal full thickness injury was made in the patellar groove using a custom made device in which the length of a 26G needle was limited by a glass bead (injured knee). The patellar dislocation was then reduced and the joint capsule and the skin sutured in separate layers. The mice were then allowed to walk freely in standard cages and maintained on free diet. Control mice were subjected to the arthrotomy and to the patellar dislocation, but no cartilage injury was made (sham operated controls). The animals were killed at different time-points and the knees dissected for histological and histochemical analysis. The same procedure has been performed in 9 month old mice of the same strain and sex and produced analogous results.

## Results

### An *in vitro *model of mechanical injury to adult human articular cartilage

To screen for signaling molecules regulated by mechanical damage in adult human articular cartilage we have adapted an *in vitro *model of mechanical cartilage injury (Figure 1a). Under our experimental conditions, uninjured explants preserved metachromatic staining with safranin O (Figure 1b) and toluidine blue (not shown) for at least 6 days. To validate this *in vitro *assay, we tested if we could detect in this injury model upregulation of metalloproteinase (MMP)-3 and MMP-13, as has been reported following mechanical cartilage injury *in vitro *and *in vivo *[26-28]. Under our experimental conditions, expression of *MMP-3 *and *MMP-13 *mRNA was significantly upregulated in the injured explants of each pair at the day 1 (*p *< 0.05) and day 6 (*p *< 0.01 for *MMP-3*; *p *< 0.05 for *MMP-13*) time points (Figure 1c,d).

### Morphogenetic pathways modulated by mechanical injury

**Figure 2:**
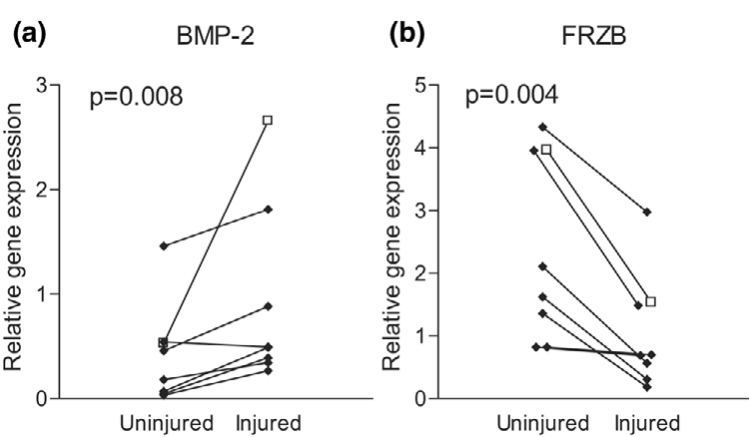
Differential expression of *bone morphogenetic protein *(*BMP*)-2 and *FRZB *mRNA following mechanical injury. **(a) ***BMP-2 *mRNA was significantly upregulated and **(b) ***FRZB *mRNA significantly down-regulated in most injured samples compared to uninjured adjacent controls. Values were calculated using a standard curve and normalized for the housekeeping *β actin *gene. Diamonds indicate samples from preserved areas from joints affected by osteoarthritis; open squares indicate the sample pair from healthy cartilage.

We then performed a differential gene expression analysis by Q-PCR, comparing the injured versus the paired uninjured explants by focusing on molecular pathways known to play a role in embryonic skeletogenesis and in the repair of other tissues. We detected statistically highly significant upregulation of *BMP-2 *mRNA (Figure 2a) and down-regulation of the secreted WNT inhibitor *FRZB *mRNA (Figure 2b) 1 day after injury (*p *< 0.01).

### Mechanical injury is associated with modulation of the BMP pathway

**Figure 3:**
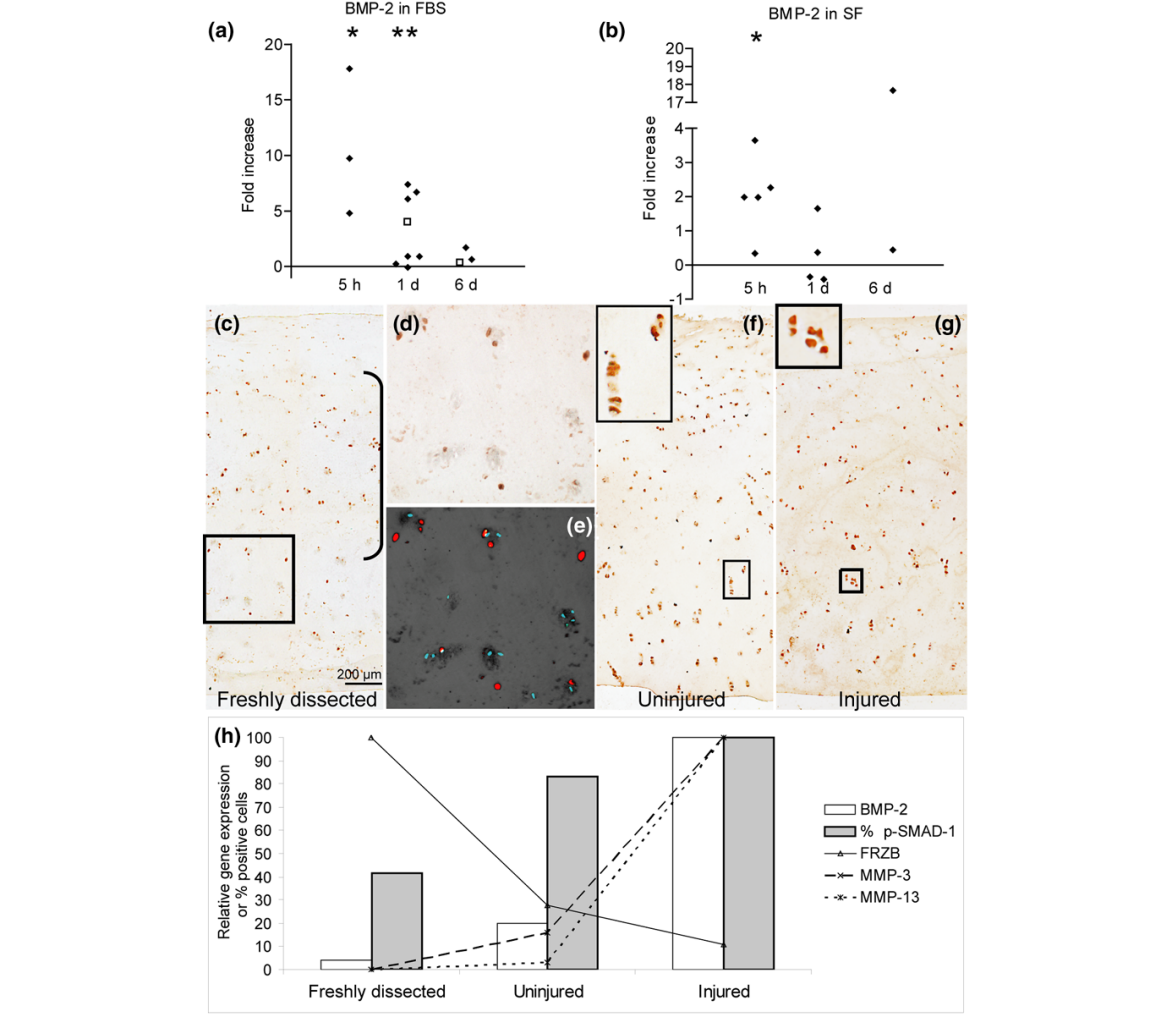
Activation of the bone morphogenetic protein (BMP) signaling pathway. **(a,b) **Time course of the differential expression of *BMP-2 *mRNA in injured versus uninjured explants in **(a) **the presence or **(b) **the absence of fetal bovine serum (FBS) in the culture medium. Values are normalized for the housekeeping *β actin *gene and expressed as fold change of gene expression in the injured explants from paired uninjured controls. Diamonds indicate samples from preserved areas from joints affected by osteoarthritis; open squares indicate the sample pair from healthy cartilage. **(c-g) **Immunostaining for phosphorylated SMAD-1/-5 in: **(c) **freshly dissected normal cartilage; **(g) **the adjacent injured explant at day 1 after injury; **(f) **and the adjacent uninjured control at the same time-point. **(d) **Larger magnification of the area shown in the square in (c). In the freshly dissected sample, phosphorylated SMAD-1/-5-positive cells were detected predominantly in the intermediate layer indicated by the bracket in (c). **(e) **Image obtained by false coloring in red the image in (d) and superimposing it on the fluorescent image in the blue channel documenting the nuclear DAPI counterstain. The DAB precipitate in the phosphorylated SMAD-1/-5-positive cells quenched the DAPI fluorescence and, therefore, in this panel, phosphorylated SMAD-1/-5-positive cells appear red and the nuclei of negative cells appear blue. The top insets in (f,g) are large magnifications of the corresponding squared areas. **(h) **A graphic summary of the proportion of phospho-SMAD-1/-5-positive cells and the expression of *BMP-2*, *FRZB*, *metalloproteinase *(*MMP*)*-3 *and *MMP-13 *mRNAs in this experiment with normal adult human articular cartilage. Values are expressed as: percent of positive cells for phospho-SMAD-1/-5; relative gene expression normalized for the housekeeping *β actin *gene; percent of the day 6 time point for *BMP-2*, *MMP-3 *and *MMP-13 *mRNA; and percent of the freshly dissected cartilage for *FRZB*. *p < 0.05; **p < 0.01. D, day(s); H, hours; SF, serum free medium.

To determine the temporal window of *BMP-2 *mRNA regulation, we performed a time course gene expression analysis at 5 hours, 1 day, and 6 days after injury. Statistically significant (*p *< 0.05) upregulation of *BMP-2 *was detected already 5 hours after wounding and tended to subside within 6 days (Figure 3a). Similar results were obtained in the absence of serum, where a statistically significant (*p *< 0.05) upregulation of *BMP-2 *mRNA was present 5 hour after injury (Figure 3b), indicating that, under our experimental conditions, the regulation of *BMP-2 *expression in response to mechanical injury is not serum dependent.

**Figure 4:**
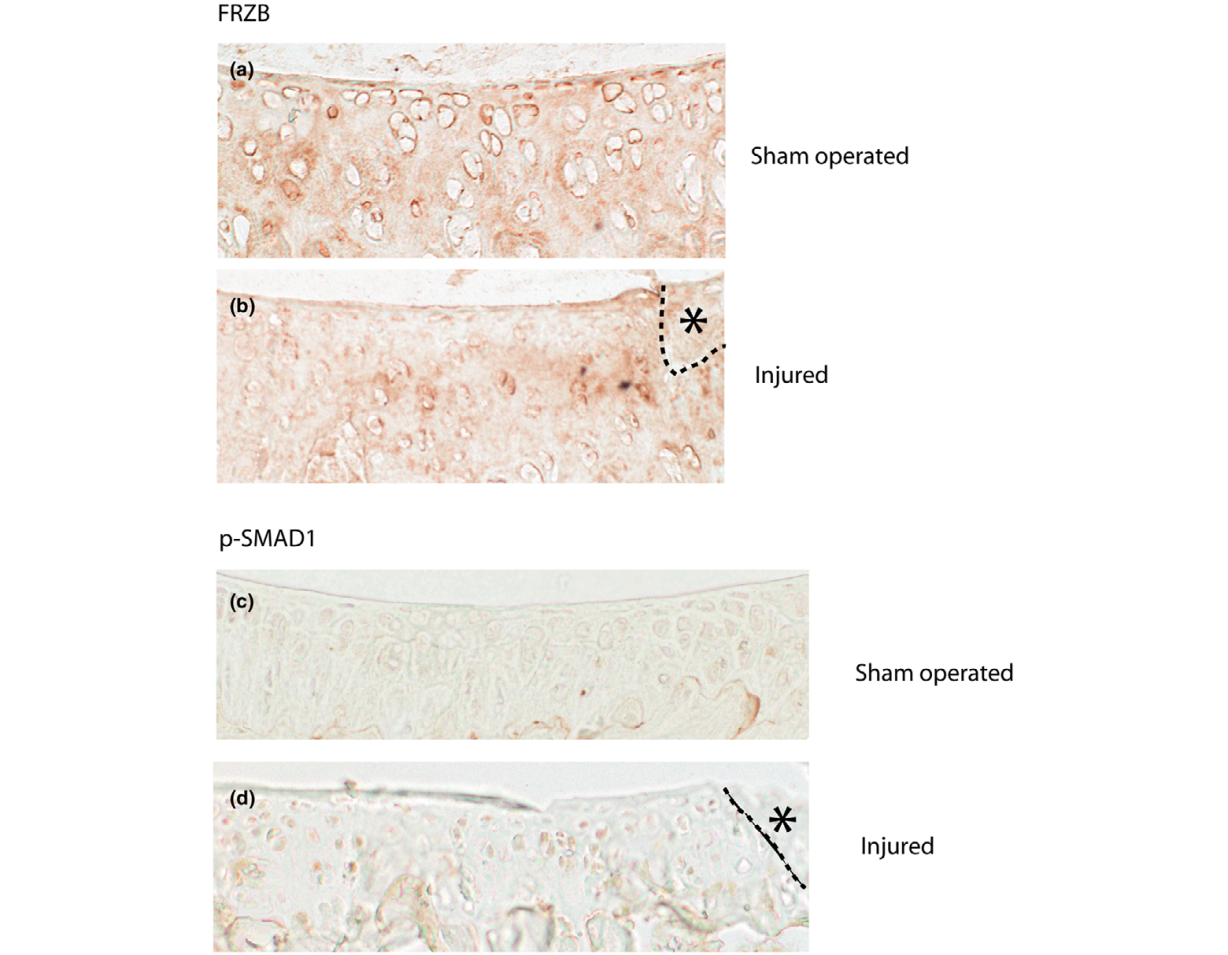
A figure showing modulation of the BMP and WNT pathway after mechanical injury *in vivo *in mice. Modulation of BMP and WNT pathway after mechanical injury in vivo in mice. 7 week old C57BL/6 male mice were challenged in a model of joint surface injury in vivo. In this model the knee joint surface is exposed by medial para-patellar arthrotomy and lateral patellar dislocation. A full thickness injury is made in the patellar groove using a custom made device in which the length of a 26G needle is limited by a glass bead (injured knee), or left uninjured (sham operated control). In either case the patellar dislocation is then reduced and the joint capsule and the skin sutured in separate layers and the mice allowed to walk freely. The animals were killed at different time-points for histological and histochemical analysis. A-B immunohistochemistry for FRZB in sham operated (A) and injured (B) articular cartilage 1 day after the operation. C-D immunohistochemistry for phosphorylated SMAD-1 in sham operated (A) and injured (B) articular cartilage 6 days after the operation. The asterisk indicates the site of injury (occupied by debris). The dashed line indicates the margin of the injury site.

To test whether the adult cartilage tissue is itself a target of BMP signaling, we performed immunohistochemistry using an antibody that recognizes the phosphorylated form of the MAD homology domain 2 of SMAD-1 and SMAD-5 [25]. In the explant pair obtained from normal articular cartilage, we detected phospho-SMAD-1/-5-positive chondrocytes in all cartilage layers in the uninjured as well as the injured explants (83% in the uninjured explant versus 100% in the injured) (Figure 3f–h). However, in adjacent uncultured freshly dissected articular cartilage, the proportion of phospho-SMAD-1/-5-positive cells was 41%, with nearly all positive cells localized in the intermediate layer (Figure 3c–e,h). These results suggest that the dissection of the cartilage explants from the joints may be associated with a molecular response to wounding, which the resting period in culture reverted only partially. Consistent with this hypothesis, *BMP-2*, *MMP-3*, and *MMP-13 *mRNA levels were lowest in the freshly dissected cartilage, intermediate in the uninjured cultured explant, and highest in the injured explant, while *FRZB *mRNA levels had an opposite trend. Similar results for the proportions of phospho-SMAD-1/-5-positive cells were found in injured and uninjured cartilage explants from OA cartilage. Finally, SMAD-1/5 phosphorylation was confirmed in vivo in a mouse model of mechanical joint surface injury (Figure 4). Full characterization of this model represents an ongoing effort in our laboratory.

### Activation of the WNT pathway following cartilage mechanical injury

**Figure 5:**
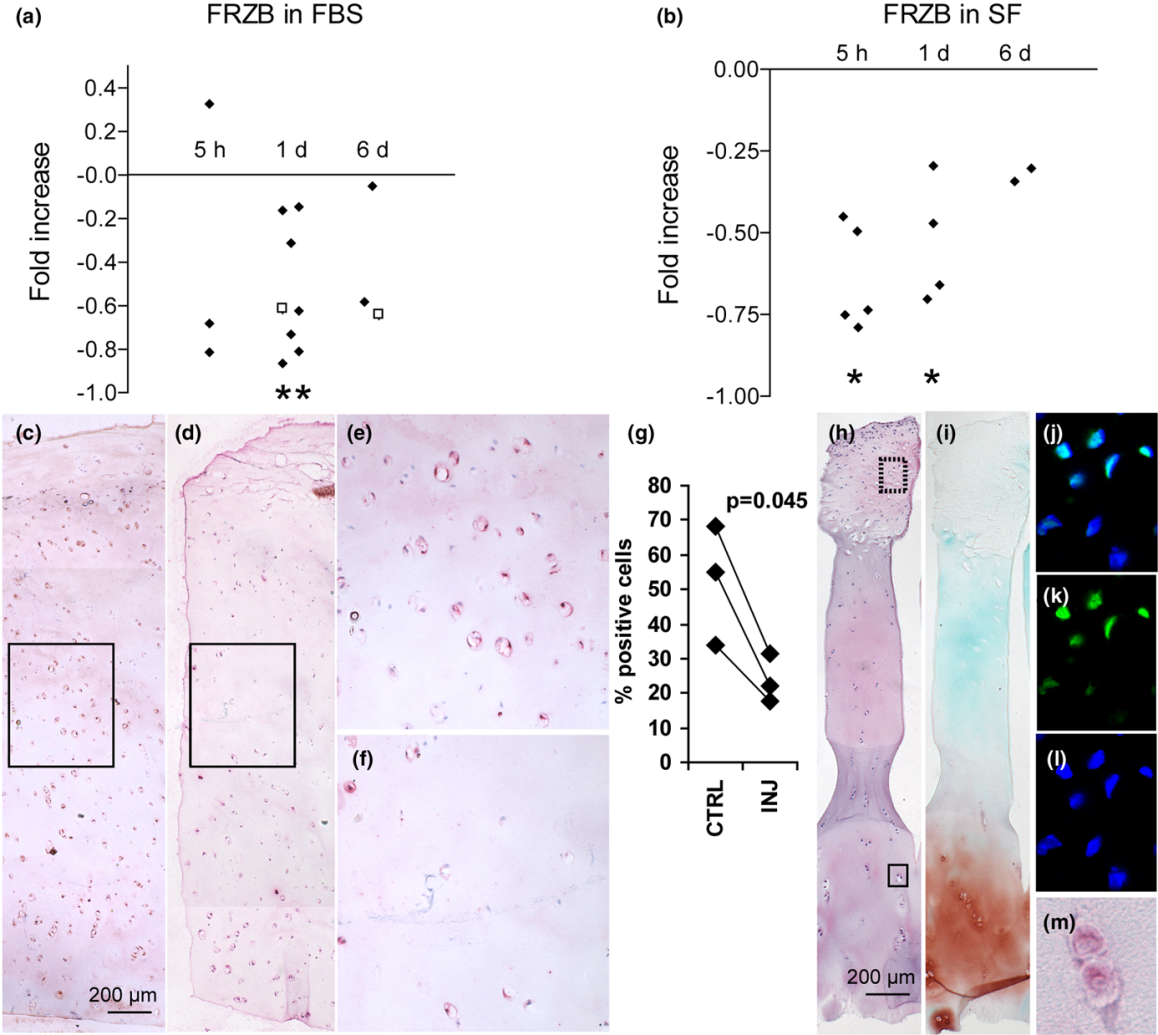
Components of the canonical WNT pathway in adult human articular cartilage. **(a,b) **Time course of the differential expression of *FRZB *mRNA in injured versus uninjured explants in **(a) **the presence or **(b) **the absence of fetal bovine serum (FBS) in the culture medium. Values were calculated using a standard curve, normalized for the housekeeping *β actin *gene and expressed as fold change of gene expression in the injured explants from paired uninjured controls. Diamonds indicate samples from preserved areas from joints affected by osteoarthritis; open squares indicate sample pairs from healthy cartilage. **(c-f) **Immunohistochemical staining for *FRZB *protein (red) in **(c) **uninjured and **(d) **injured explants at the day 1 time point. Haematoxylin was used as a nuclear counterstain. **(e,f) **Larger magnifications of the boxed areas in (c) and (d), respectively. **(g) **Percentage of FRZB-positive cells in injured explants and in the paired uninjured controls from 3 independent donors as evaluated by immunohistochemistry. **(h) **Haematoxylin-eosin and **(i) **safranin O stainings of an explant with a relatively high degree of osteoarthritis (modified Mankin score 5). **(j-m) **Immunostaining for β catenin in parallel, non-consecutive sections of (h) and (i). **(j-l) **Indirect immunofluorescence stainings for β catenin from a parallel section in the area of (h) boxed with the dashed line (top). **(k) **β catenin (green). **(l) **DAPI counterstain of the same section (blue). **(j) **The superimposition of (k) and (l). In this tissue, which is commonly called pannus, there were cells with a nuclear localization of β catenin. **(m) **Immunohistochemistry showing the cytoplasmic localization of β catenin in chondrocytes of the basal layer (area in (h) boxed with a solid line). *p < 0.05; **p < 0.01. D, day(s); H, hours; SF, serum free Medium.

In a time course analysis, *FRZB *mRNA was already down-regulated in some but not all explant pairs 5 hours after injury (Figure 5a). Similar results were obtained with serum free culture conditions (Figure 5b), thereby demonstrating that, under our experimental conditions, *FRZB *mRNA regulation in response to mechanical injury was not dependent on the presence of FBS in the culture medium. Statistical analysis confirmed a highly significant difference (*p *< 0.01) at the day 1 time point in the presence of FBS and a significant difference (*p *< 0.05) at the 5 hour and day 1 time points in the absence of serum. At the protein level, FRZB was present in both injured and uninjured explants as evaluated by immunohistochemistry (Figure 5c–f). The proportion of FRZB positive cells was significantly lower (*p *< 0.05) in the injured explant in three independent explant pairs, confirming at the protein level the down-regulation of FRZB expression in the injured explants (Figure 5g). Downregulation of FRZB was confirmed at protein level *in vivo *in a mouse model of joint surface injury (Figure 4). The down-regulation of the secreted inhibitor *FRZB *suggests de-repression of WNT signaling. Thus, we next investigated whether the expression of components of the WNT pathway that are present in cartilage during mouse embryonic development [29,30] is maintained in adult human articular cartilage. We detected mRNA encoding WNT ligands (*WNT-1*, *WNT-5a*, *WNT-5b*, *WNT-9a/14*, and *WNT16*), receptors (*FRZD-1 *and *FRZD-7*), intracellular mediators such as β-catenin, and downstream transcription factors such as *TCF *and *LEF-1 *(data not shown). The presence of β-catenin was also confirmed at the protein level (Figure 5h–m).

**Figure 6:**
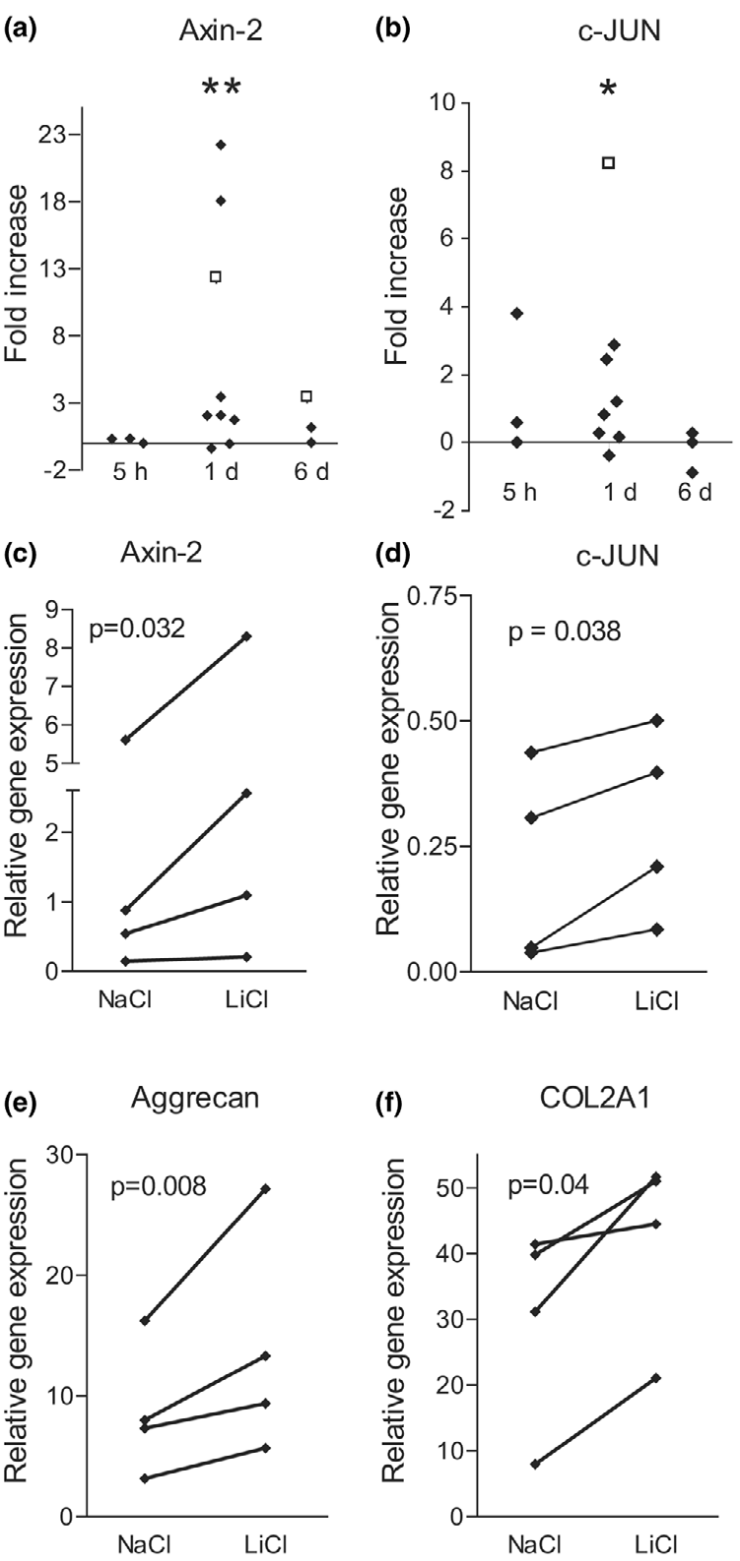
Activation of the WNT/β catenin canonical pathway following mechanical injury. **(a) ***Axin-2 *and **(b) ***c-JUN *mRNAs, two known transcriptional targets of the WNT/β catenin canonical pathway, were upregulated 1 day after injury compared to uninjured controls. **(c-f) **Paired cartilage explants were cultured in the presence of either 10 mM LiCl or 10 mM NaCl for 1 day and then terminated for gene expression analysis by quantitative real time PCR. Culture in the presence of LiCl induced the upregulation of *axin-2 *(c) and *c-JUN *(d) mRNAs, thereby confirming that these two genes are targets of the WNT/β catenin canonical pathway in this experimental system. LiCl treatment also upregulated aggrecan and COL2A1 mRNA (e,f). **p < 0.01. D, day(s); h, hours.

We then investigated whether mechanical injury resulted in a net activation of the canonical WNT pathway by performing gene expression analysis of the WNT target genes *Axin-2 *[31] and *c-JUN *[32,33]. Consistent with our hypothesis and with the activation of the WNT/β-catenin signaling pathway, *Axin-2 *mRNA was upregulated 1 day after mechanical injury (Figure 6a), with a statistically highly significant difference (*p *< 0.01). *c-JUN *[32,33] was also significantly (*p *< 0.05) upregulated in the injured explants, although to a lesser extent then *Axin-2 *(Figure 6b). To confirm that *Axin-2 *and *c-JUN *mRNA are WNT targets in adult articular cartilage and under our experimental conditions, we monitored the expression of these genes after treatment with 10 mM LiCl, an inhibitor of GSK-3 and, therefore, an activator of the β catenin-dependent WNT signaling pathway [34]. The expression of *Axin-2 *and *c-JUN *was consistently and significantly (*p *< 0.05) upregulated in the LiCl-treated explants compared with the paired control explants treated with NaCl (Figure 6c,d). To test the effects of the activation of the canonical WNT pathway in adult human articular cartilage, we determined the expression of the cartilage markers *COL2A1 *and *Aggrecan *in LiCl treated and control cultures. Under our experimental conditions, LiCl treatment significantly (*p *< 0.05) upregulated *COL2A1 *and *Aggrecan *mRNA, suggesting an anabolic effect (Figure 6e,f).

## Discussion

The articular cartilage of adult individuals is commonly regarded as a passive target of different pathogenic elements, such as mechanical wear and inflammation, leading to cartilage matrix breakdown and loss of chondrocytes. However, acute, small, full thickness JSDs appear to have repair capacity in animals and humans, especially in young individuals [2,3,5-8]. Repair of full thickness JSDs involves coordination of patterning and tissue maturation that recapitulates some aspects of embryonic skeletal development [6], thereby requiring morphogenetic signaling. Here we have tested the hypothesis that the injured articular cartilage may be a source of morphogenetic signals activated by damage. To this end we have used an *ex vivo *model to investigate the modulation of gene expression induced by mechanical injury to adult human articular cartilage explants. We have detected upregulation of *BMP-2 *mRNA after injury. Several factors can determine activation of BMP signaling independently of the expression of one ligand, including secretion and solubility of the ligand(s), its/their binding to matrix molecules, the presence of secreted or intracellular inhibitors and receptor regulation [35]. Our data showing phosphorylation of SMAD-1/-5 suggest activation of BMP signaling.

BMPs elicit a well-documented anabolic response on cartilage explants [20], and genetic evidence has been provided that the BMP pathway is needed for joint homeostasis in adulthood [36]. Indeed, targeted deletion of the gene encoding BMP receptor 1A in the articular cartilage in mice results in joint surface degeneration resembling OA [36]. In addition, BMPs have been shown to regulate recruitment of chondroprogenitors [37], synthesis of cartilage matrix, and endochondral bone formation [20] during embryonic skeletogenesis. Finally, the expression of *BMP-2 *mRNA is associated with the capacity of *in vitro *expanded adult human articular chondrocytes to form stable cartilage *in vivo*, resistant to vascular invasion and endochondral bone formation [23]. Therefore, the recruitment of progenitor cells, the regulation of endochondral bone formation and cartilage extracellular matrix synthesis, as well as the preservation of the phenotypic stability of articular chondrocytes are all potential roles of BMP signaling in JSD repair. However, it must be underscored that BMP signaling also plays a part in the pathogenesis of joint diseases such as osteophyte formation in OA [38] and enthesopathy [39]. Finally, upregulation of BMP-2 has already been reported following exposure of cartilage explants to interleukin 1 and tumor necrosis factor alpha [40]. It is possible, therefore, that upregulation of BMP-2 may represent a response of the articular cartilage to different types of injuries.

In addition to the upregulation of *BMP-2 *mRNA, we have documented a consistent injury-associated down-regulation of the secreted WNT inhibitor FRZB, suggesting de-repression of the WNT signaling pathway. Consistently, we have detected, in the injured explants, upregulation of mRNA encoding the WNT/β catenin transcriptional targets *Axin-2 *and *c-JUN*. The WNT signaling pathway can be regulated at multiple levels [22] and, therefore, our experimental setup does not allow determining whether the decreased expression of *FRZB *mRNA is responsible for the detected upregulation of the WNT/β catenin target genes. Nevertheless, the functional importance of the regulation of FRZB expression in the context of joint homeostasis is underscored by the observation that a single nucleotide polymorphism causing loss of function of the *FRZB *gene product is associated with hip OA in humans [41].

The function of WNT signaling in the context of joint surface defect repair is still poorly understood. Studies on embryonic tissues indicate that the activation of the canonical β catenin pathway plays an important role in joint specification [30,42] and in the regulation of chondrocyte differentiation inhibiting chondrogenesis in immature mesenchymal cells and enhancing terminal differentiation in mature chondrocytes [29,32]. However, while the data in embryonic tissues suggest a general inhibitory effect of canonical WNT signaling on chondrogenesis, in experimental models utilizing adult cells, the activation of the β catenin-dependent canonical WNT pathway, under specific experimental conditions, rather appears to promote chondrogenesis and cartilage differentiation [43-45]. This is in line with our findings that adult human articular cartilage explants cultured in the presence of LiCl upregulate *COL2A1 *and *aggrecan *mRNA. Since in other organ systems WNTs are involved in supporting repair processes by maintaining a stem cell pool and specifying cell fates [19,46,47], it is tempting to speculate that the canonical WNT pathway would play a similar function in the repair of osteochondral defects. Finally, there is also evidence that WNTs, at least through the non-canonical pathway, may be implicated in joint inflammation and may be detrimental for cartilage integrity [48]. The most likely interpretation of these apparently contrasting data is that a tight regulation of the WNT and the BMP pathways is necessary for proper joint homeostasis and repair and that, in postnatal life, the same mechanisms that are set into action to support repair may also play a pathogenic role when de-regulated or when restoration of homeostasis fails. In this regard, it is interesting that gain or loss of function of β catenin in the developing skeleton both result in severe chondrodysplasia, although through different mechanisms [49].

We have encountered a high variability in the molecular responses to injury in different pairs of cartilage explants. This variability can be explained by the heterogeneity of tissues from patient to patient, and by our inability to obtain adequately 'homogeneous' preparation of the explants. Analogous variability has been reported in the molecular response of cartilage explants to inflammatory cytokines [50]. Indeed, the variability in the molecular response to injury could be a factor contributing to the variability in the clinical outcome of untreated acute articular cartilage injuries.

In some experiments, the differences in gene expression were of small magnitude. However, we have observed a reproducible upregulation of WNT reporter genes, including *Axin-2*, following injury or LiCl treatment, which indicate that the modulation of the wnt signaling was sufficient to induce a transcriptional response. *Axin-2 *upregulation of approximately the same magnitude was reported to be associated with increased bone mass in osteoporotic *lrp5*^-/- ^mice following oral administration of LiCl [51]. Remarkably, the plasma levels of LiCl achieved in that study were only 0.4 to 0.5 mM, which are insufficient to trigger detectable wnt responses in the classic assays such as β catenin nuclear localization or activation of the TOP-FLASH reporter. It is reasonable that this magnitude of wnt activation in adult animals is probably more physiological than that achieved in overexpression experiments [51]. Indeed, in postnatal life, morphogenetic events take place at a much lower rate than in embryonic development and, therefore, slight changes in the balance of the morphogenetic pathways can result in significant biological effects.

The *in vitro *culture conditions may influence the molecular response to injury, potentially introducing artifacts. However, the reproducibility of *FRZB *and *BMP-2 *mRNA regulation in response to damage regardless of the presence of serum in the culture medium suggests that this response is largely not dependent on culture conditions. In addition, it is possible that the response to injury *in vivo *will be more vibrant than that *in vitro *because the resting period does not appear to be sufficient to completely reverse the response due to the initial dissection of the explants. In this respect, Vincent and colleagues [52] reported rapid phosphorylation of ERK following dissection of porcine articular cartilage explants, which was completely reverted after 48 hours of "resting" in culture. In our study, the modulation of *BMP-2 *and *FRZB *mRNA appear to last longer than 48 hours. This is also supported by the analysis of the sample in Figure 3h, in which the expression levels of all molecules tested and the number of phospho-SMAD-1/-5-positive cells in the rested explant were intermediate between the freshly dissected explant and the explant re-injured after the resting period. Most importantly, we have shown phosphorylation of SMAD-1/-5 and downregulation of FRZB expression *in vivo *in a mouse model of joint surface injury (Figure 4) not only confirming our data *in vivo*, but also suggesting that such mechanisms are evolutionarily conserved. Functional studies are being performed to evaluate the role of these molecular mechanisms in the context of cartilage damage and repair. Full characterization of this model is an ongoing effort in our laboratory.

Injuries to the articular cartilage result in activation of the bone marrow and subchondral bone remodeling [53], suggesting the presence of molecular signals that are released and target the neighboring tissues. We have demonstrated that mechanical injury *in vitro *can elicit the activation of two of the most important signaling pathways involved in embryonic skeletogenesis and joint morphogenesis, suggesting that the articular cartilage is capable of triggering a signaling machinery that may play a role in joint surface repair.

Although several risk factors for OA have been identified, including the nature and entity of the injury, age, genetic predisposition, and joint congruity, it is still not clear why some individuals can efficiently repair JSDs while some others will develop chronic symptomatic lesions requiring surgical intervention and possibly evolving into OA [1]. Failure of repair signaling may contribute to evolution towards OA. Our data suggest that morphogenetic pathways are transiently activated early following acute injury, as has been reported in other organ systems [19]. Insufficient or untimely activation of this machinery may result in repair failure. It is important, therefore, to study these events in a temporally dynamic fashion, and it is possible that the early post-traumatic signals may be critical for the final repair outcome. Understanding the molecular mechanisms of repair may help us define a more focused indication for biological JSD repair. On the other hand, the modulation of these signaling pathways (e.g., by controlled release of bioactive molecules from scaffolding biomaterials) may complement the available tissue engineering approaches to enhance specific aspects of repair. Finally, the persistence in adulthood of locally residing stem cells within several joint tissues, including bone marrow [54], synovial membrane [16], periosteum [13], and articular cartilage [12,14,15,18], opens the possibility to recruit and guide these cells locally using appropriate molecular signals to enhance repair. This would circumvent a number of problems associated with *ex vivo *cell manipulation, including phenotypic instability, high costs, non-optimal consistency, and complex regulation of the cellular products [55].

## Conclusion

Our data show modulation of the WNT and BMP signaling pathways in adult human and mouse articular cartilage following mechanical injury *in vitro *and *in vivo*. These molecular events may contribute to trigger or support a repair response and failure to promptly activate these reparative signals may contribute to poor repair and poor clinical outcome. Hence, activation of the WNT and BMP pathways in response to injury may represent a prognostic marker and at the same time a therapeutic target to enhance the early response of the joint surface to acute injury.

## Abbreviations

BMP = bone morphogenetic protein; glycogen synthase kinase 3 = GSK-3; DAPI = 49,6-diamidino-2-phenylindole; FBS = fetal bovine serum; JSD = joint surface defect; MMP = metalloproteinase; OA = osteoarthritis; Q-PCR = quantitative real time PCR; RT-PCR = reverse transcription PCR; TBST = tris buffered saline; TCF/LEF = T-cell factor/lymphoid enhancer factor.

## Competing interests

The authors declare that they have no competing interests.

## Authors' contributions

FD designed the study, performed the experiments and drafted the manuscript. CD was involved in the study design, in data interpretation, and drafting the manuscript. NE contributed to immunohistochemical stainings for FRZB. FB contributed to the optimization of the phospho-SMAD-1 staining. TM critically revised the manuscript for important intellectual content. JO critically revised the manuscript for important intellectual content. CP was involved in the study design, interpretation of the results and has critically reviewed the manuscript.
